# Excretion of SARS-CoV-2 RNA in feces has no prognostic benefit in the outcome of COVID-19: A clinical and immunological study

**DOI:** 10.17305/bb.2024.10176

**Published:** 2024-08-01

**Authors:** Božo Šušak, Monika Dalmatin-Dragišić, Luka Laura, Vinka Mikulić, Katarina Nakić, Ivanka Mikulić, Ilija Brizić, Jurica Arapović, Maja Arapović

**Affiliations:** 1 Department of Infectious Diseases, University Clinical Hospital Mostar, Mostar, Bosnia and Herzegovina; 2 School of Medicine, University of Mostar, Mostar, Bosnia and Herzegovina; 3 Faculty of Pharmacy, University of Mostar, Mostar, Bosnia and Herzegovina; 4 Department of Laboratory Diagnostics, University Clinical Hospital Mostar, Mostar, Bosnia and Herzegovina; 5 Center for Proteomics, Faculty of Medicine, University of Rijeka, Rijeka, Croatia

**Keywords:** Coronavirus disease 2019 (COVID-19), severe acute respiratory syndrome coronavirus 2 (SARS-CoV-2), COVID-19 testing, immunoglobulin G, vaccination, viral load, feces, signs and symptoms, patient outcome

## Abstract

This study explores the correlation between immunological and clinical characteristics in coronavirus disease 2019 (COVID-19) patients with detectable severe acute respiratory syndrome coronavirus 2 (SARS-CoV-2) RNA in feces, analyzing data from 251 patients admitted to Mostar University Clinical Hospital (UCH) from December 2021 to January 2022. Methods involved reverse transcription quantitative polymerase chain reaction (RT-qPCR) from nasopharyngeal (NP) swabs and feces, alongside serological tests for anti-SARS-CoV-2 spike IgGs. Demographic and clinical data were collected through questionnaires and medical records. The data analyses were performed using SPSS statistical software. Death occurred in 53 patients (21.1%, *P* < 0.001), mostly in the elderly (47/53, 88.7%, *P* ═ 0.001) and immunocompromised (19/53, 35.8%, *P* ═ 0.05), particularly those developing acute respiratory insufficiency (ARI) (46/53, 86.8%, *P* ═ 0.004), and severe/critical disease (46/53, 86.8%, *P* ═ 0.002). Among the patients with positive anti-SARS-CoV-2 IgG antibodies (86/251, 34.3%, *P* < 0.001), 41 (47.7%) were vaccinated and 45 (52.3%) unvaccinated (*P* ═ 0.666), showing no significant differences in clinical outcomes or mortality. Unvaccinated patients with a negative antibody titer had a higher incidence of ARI (96/123, 78%, *P* ═ 0.029) and intensive care unit (ICU) admission (22/123, 17.9%, *P* ═ 0.026), than those with a positive antibody titer. Forty-seven (62.7%) patients, out of the 75 hospitalized who provided a feces sample, were positive for SARS-CoV-2 RNA (*P* ═ 0.028), without statistical differences between fecal SARS-CoV-2 positive and negative groups regarding vaccination status (15/47, 31.9%, *P* ═ 0.493), antibody status (18/47, 38.3%, *P* ═ 0.628), or death outcome (5/47, 10.6%, *P* ═ 0.706). In conclusion, unvaccinated hospitalized patients with a severe COVID-19 presentation and a negative anti-spike SARS-CoV-2 IgG titer had adverse outcomes more frequently. This suggests cautious consideration for the diagnostic use of fecal samples compared to NP swabs.

## Introduction

Severe acute respiratory syndrome coronavirus 2 (SARS-CoV-2), the cause of coronavirus disease 2019 (COVID-19), remains a threat to global health, with various genetic lineages emerging and circulating globally [1]. The gold standard for the diagnosis of COVID-19 is reverse transcription quantitative polymerase chain reaction (RT-qPCR) which determines the presence of genomic material of SARS-CoV-2 in samples from different origins. The viral RNA is commonly detected in nasopharyngeal (NP) swabs, but it can also be detected in sputum, urine, lung, serum, plasma, and feces samples [2, 3]. The value of the cycle threshold (Ct value) determined by RT-qPCR is inversely proportional to viral load [4]. A prior study showed that the viral load from NP swabs was the highest at the beginning of the symptoms or a few days after, followed by a significant decline two weeks after the onset of symptoms [5], whereas viral loads in feces samples usually peak later on, indicating later viral clearance in feces samples [6]. Higher viral load in NP swabs positively correlates to virus infectivity and COVID-19 severity, while SARS-CoV-2 RNA shedding duration and feces viral load dynamics require further research, including the virus transmissibility and clinical significance [5, 6].

In addition to RT-qPCR testing, serological tests are used to identify currently or previously SARS-CoV-2-infected individuals, based on the detection of specific antibodies (IgM, IgG, IgA immunoglobulins) to SARS-CoV-2 antigens [7]. The IgG antibodies have affinity to its antigen and a high efficiency for pathogen neutralization resulting in mostly systemic protection against COVID-19 [8]. They appear later in the immune response and are associated with long-term immunity following infection or vaccination [9]. Consequently, the specific antibody titer is an accurate method to detect neutralizing antibodies from recently resolved or past infections. Antibody titers are decreasing following infection resolution [10]. The neutralizing antibodies are stalling infection if antibody titers are at an optimal level. Accordingly, one of the main goals of vaccination is the induction of antibody titers at a similar or higher level as in convalescent individuals. In addition, vaccination can expand and improve antibody clonal lineages induced by previous infection, thus preventing COVID-19 severity against novel virus strains [11].

Patients in our hospital were tested by NP swab, although there is evidence that SARS-CoV-2 RNA can be detected in samples from different origins [12]. Members of the coronavirus family, SARS-CoV and Middle East respiratory syndrome coronavirus (MERS-CoV), are known to be excreted in the feces of infected patients, suggesting a potential for fecal-oral transmission [13]. Similar to these viruses, SARS-CoV-2 was also found in the feces of a significant number of patients with COVID-19 [14]. Recent studies have shown no correlation between the excretion of SARS-CoV-2 RNA in the feces and the outcome of COVID-19 [15, 16].

This study aimed to determine the association of fecal virus excretion with immunological and clinical characteristics, as well as clinical outcomes of COVID-19 patients.

## Materials and methods

### Study design

A total of 251 SARS-CoV-2-positive participants admitted to the COVID department of the University Clinical Hospital (UCH) Mostar between December 2021 and January 2022 were enrolled in the study. The sociodemographic and clinical data were collected from questionnaires and medical records.

### Reverse transcription quantitative polymerase chain reaction (RT-qPCR)

NP swabs were collected from patients at hospitalization between the fifth and seventh day of symptom onset and were routinely analyzed using RT-qPCR. The criteria for SARS-CoV-2/COVID-19 positive tests were Ct values ≤ 38 for both the RNA-dependent RNA polymerase (RdRp) and nucleocapsid protein (N) genes, according to the manufacturer’s instructions. Based on PCR test results and clinical status, patients were hospitalized and consequently included in our study.

Four SARS-CoV-2 genes were analyzed after the swabs underwent extra processing: RdRp, N, non-structural protein 14 (nsp14), and envelope protein (E). Using an RNA extraction kit and reverse transcription, RNA was transcribed into complementary DNA (cDNA) using a one-step RT-qPCR assay (qScript XLT One-Step RT-qPCR ToughMix, Quanta Bio), following the manufacturer’s protocol. Eurofins Genomics (Vienna, Austria) produced and supplied the primers and probes. The reaction was carried out by a magnetic induction cycler (MIC PCR, Bio Molecular Systems) and the results were interpreted as previously reported [17].

Feces samples were collected within the first three days of hospitalization, and analyzed by RT-qPCR after short-term storage at +4 ^∘^C. 30 mg of feces was suspended in 600 µL of lysis buffer (RLys buffer) for every sample to obtain 5% w/v suspension. Feces suspensions were then homogenized by vortexing for 60 s, followed by centrifugation for 2 min at 15,000 rotations per minute. Supernatants were then used as starting points for RNA extraction, which was performed by using an RNA extraction kit for tissues (EXTRACTME total RNA kit, BLIRT, S.A.), according to the manufacturer’s protocols. Obtained RNA eluates were then analyzed for four genes (RdRP, E, N, and nsp14) by the same protocol that was previously described for NP swabs. Confirmed-positive samples were used as positive controls for all RT-qPCR reactions, while no-template controls served as negative controls.

### Serological testing

Serum samples, after routine biochemical processing, were stored at –20 ^∘^C until analysis. The serum concentration of SARS-CoV-2 IgGs was quantitatively determined using the SARS-CoV-2 IgG reagent kit (REF-11207376, Siemens Healthcare Diagnostic Inc., USA) on the ADVIA Centaur XPT analyzer (Siemens Healthcare Diagnostics Inc., USA), as described previously [18].

### Ethical statement

The study was conducted in accordance with the ethical standards stated in the 1964 Declaration of Helsinki and its subsequent amendments. Ethical approval was acquired from the Ethical Committee at UCH Mostar, number 1035/21.

### Statistical analysis

Descriptive and analytical statistical methods were used in data processing. Data were presented as mean ± SD or median and number (percentage) for categorical variables. A chi-squared or Fisher’s exact test was used for the analysis of categorical data. Pearson (*r*) and Spearman (Rho) tests were used for the correlation of continuous and discrete data, respectively. We first converted the continuous variable into categorical variables using the median and quartile analysis, for the correlation test, and then the Spearman test with a two-tailed test of significance was performed for the correlation. All statistical analyses were performed using SPSS version 23.0 software (SPSS Inc., Chicago, IL, USA).

## Results

The median age of patients included in this study was 72 (25–92) years, and 154 patients (61.4%) were male. Most of the patients had previous comorbidities, most often arterial hypertension and other cardiovascular diseases, as well as diabetes mellitus (78.5%, *P* < 0.001) (Table 1). Sixty-six patients (30.7%, *P* < 0.001) were vaccinated with two doses of COVID-19 vaccines, and nine of them (4.9%, *P* ═ 0.001) reported a previous COVID-19 infection. The majority of the patients developed pneumonia before or during hospitalization (90%, *P* < 0.001). Out of a total of 251 patients, 178 (70.9%) developed acute respiratory insufficiency (ARI), 176 (70.1%) manifested severe or critical form of COVID-19, whereas 35 (13.6%) of patients were admitted to the intensive care unit (ICU) (Table 1). Death occurred in 53 (21.1%, *P* < 0.001) of patients, mostly in the elderly (47/53, 88.7%, *P* ═ 0.001), immunocompromised (19/53, 35.8%, *P* ═ 0.05), those who developed ARI (46/53, 86.8%, *P* ═ 0.004), and with severe or critical disease (46/53, 86.8%, *P* ═ 0.002) (Table S1).

**Table 1:** Main statistical data

**Characteristics of patients, *n* ═ 251**		***n* (%)**	***P* value**
Age (years)	18–44	14 (5.6)	**<0.001**
	45–64	60 (23.9)	
	65–74	66 (26.3)	
	>75	111 (44.2)	
Gender	Male	154 (61.4)	**<0.001**
Comorbidities	Yes	197 (78.5)	**<0.001**
	Arterial hypertension	158 (62.9)	
	Diabetes mellitus	66 (26.3)	
	Cardiovascular diseases	80 (31.9)	
	Pulmonary diseases	26 (10.4)	
	Immunocompromised	63 (25.1)	
	Kidney disease	11 (4.4)	
COVID-19 status	Recovered	9 (4.9)	**<0.001**
	Naive	176 (95.1)	
Vaccination status	Vaccinated	66 (30.7)	**<0.001**
Antibodies	Titer ≥1	86 (34.3)	**<0.001**
Symptoms	Yes	238 (94.8)	**<0.001**
	Fever >38^∘^C	92 (37.4)	**<0.001**
	Cough	174 (69.3)	**<0.001**
	Dyspnea	148 (58.9)	**<0.001**
	Diarrhea	41 (16.3)	**<0.001**
	Loss of smell and taste	36 (13.5)	**<0.001**
Systolic arterial pressure (mmHg)	<90	6 (2.4)	**<0.001**
	100–140	171 (68.1)	
	>140	74 (29.5)	
GCS	<8	5 (2.0)	**<0.001**
	8–12	6 (2.4)	
	12–15	240 (95.6)	
Disease severity	Moderate	75 (29.9)	**<0.001**
	Severe	129 (51.4)	
	Critical	47 (18.7)	
Pneumonia	Yes	226 (90.0)	**<0.001**
ARI	Yes	178 (70.9)	**<0.001**
Antimicrobial therapy	Yes	95 (37.8)	**<0.001**
Corticosteroids	Yes	189 (75.3)	**0.002**
	Dexamethasone	107 (42.6)	
	Metilprednisolone	82 (32.7)	
Anticoagulant therapy	Yes	229 (91.3)	**<0.001**
	Prophylactic	166 (66.1)	
	Therapeutic	63 (25.1)	
Complications	Yes	66 (26.3)	**<0.001**
	PE	9 (3.6)	
	CVI	5 (1.9)	
	MI	8 (3.2)	
	Pneumotorax	3 (1.2)	
	Other	41 (16.3)	
ICU admission	Yes	35 (13.6)	**<0.001**
Death outcome	Yes	53 (21.1)	**<0.001**

Bold values represent statistical significance. COVID-19: Coronavirus disease 2019; GCS: Glasgow coma scale; ARI: Acute respiratory insufficiency; ICU: Intensive care unit; PE: Pulmonary embolism; CVI: Cerebrovascular insult; MI: Miocardial infarction.

### The influence of vaccination on the clinical characteristics and outcomes of COVID-19

Out of 66 vaccinated patients, 41 patients (62.1%, *P* < 0.001) had detectable SARS-CoV-2-specific IgG antibodies and 34 (51.5%, *P* ═ 0.243) had Ct values over 30, compared to the unvaccinated patients (Table 2). The majority of the vaccinated patients had comorbidities (61/66, 92.4%, *P* ═ 0.001). Vaccinated patients (54/66, 81.8%) developed pneumonia to a lower extent than unvaccinated patients (156/168, 92.9%, *P* ═ 0.017). In addition, 38 (57.6%) vaccinated patients presented with a severe/critical form of COVID-19, compared to unvaccinated patients (125/168, 74.4%, *P* ═ 0.017) (Table 2).

**Table 2:** Demographics, chronic diseases, clinical characteristics, and outcomes of COVID-19 patients regarding the vaccination

**Characteristics of patients, *n* ═ 234**		**Vaccinated, *n* (%)**	**Unvaccinated, *n* (%)**	***P* value**
Age (years)	18–44	3 (4.5)	11 (6.5)	0.361
	45–64	11 (16.7)	44 (26.2)	
	65–74	21 (31.8)	42 (25.0)	
	>75	31 (47.0)	71 (42.3)	
Gender	Male	47 (71.2)	100 (59.5)	0.101*
	Female	19 (28.8)	68 (40.5)	
Comorbidities	Yes	61 (92.4)	124 (73.8)	0.001*
	No	5 (7.6)	44 (26.2)	
Immunocompromised	Yes	19 (28.4)	38 (22.6)	0.397
	No	48 (71.6)	130 (77.4)	
Ct value	≤30	32 (48.5)	97 (57.7)	0.243*
	>30	34 (51.5)	71 (42.3)	
Feces, *n* ═ 72	Yes	14 (66.7)	30 (58.8)	0.424*
	No	7 (33.3)	21 (41.2)	
Antibodies	Positive	41 (62.1)	45 (26.8)	**<0.001**
	Negative	25 (37.9)	123 (73.2)	
Symptoms	Yes	62 (93.9)	160 (95.2)	0.744*
	No	4 (6.1)	8 (4.8)	
Fever	<38 ^∘^C	41 (62.1)	102 (60.7)	0.826
	>38 ^∘^C	25 (37.9)	66 (39.3)	
Pneumonia	Yes	54 (81.8)	156 (92.9)	0.017*
	No	12 (18.2)	12 (7.1)	
ARI	Yes	42 (63.6)	123 (73.2)	0.155*
	No	24 (36.4)	45 (26.8)	
Oxygen support	Unknown	8 (12.1)	17 (10.1)	0.313
	<7L/min	42 (63.6)	93 (55.4)	
	≥7L/min	16 (24.2)	58 (34.5)	
Corticosteroids	Yes	44 (66.7)	130 (77.4)	0.099*
	No	22 (33.3)	38 (22.6)	
ICU	Yes	8 (12.1)	24 (14.3)	0.833
	No	58 (87.9)	144 (85.7)	
Disease severity	Moderate	28 (42.4)	43 (25.6)	0.017*
	Severe/Critical	38 (57.6)	125 (74.4)	
Death outcome	Yes	15 (22.7)	33 (19.6)	0.594*
	No	51 (77.3)	135 (80.4)	

*Fisher exact test; Bold values represent statistical significance. COVID-19: Coronavirus disease 2019; Ct: Cycle threshold; ARI: Acute respiratory insufficiency; ICU: Intensive care unit; Ct: Cycle threshold.

Further analysis showed that 14 patients (21.2%) were not aware of the type of vaccine they received, 23 (34.8%) were vaccinated with mRNA vaccines (Pfizer-BioNTech/Moderna), 15 (22.7%) with adenoviral vector vaccine (Oxford-AstaZeneca/Jonhson&Jonhson), and 14 (21.2%) with whole inactivated virus COVID-19 vaccines (Sinopharm) (*P* ═ 0.327) out of 66 vaccinated patients (Table S2). Thirty-one (47%) vaccinated patients were older than 75 years (*P* ═ 0.045), majority of males (41/66, 71%, *P* ═ 0.003). There were no significant differences among patients in clinical characteristics and outcomes of COVID-19 regardless of the received vaccine type (Table S2).

### The influence of the viral load on the clinical characteristics and outcomes of COVID-19

To analyze the influence of the viral load on the clinical manifestation of the disease, we analyzed the epidemiological and clinical parameters of patients stratified by the Ct values. Our results showed that patients with lower viral load presented by Ct value over 30 (51/100, 51%, *P* < 0.001) had higher levels of virus-specific antibodies (Table 3). Immunocompromised patients had a higher viral load with 30.4% of patients with Ct ≤ 30 (42/138, *P* ═ 0.034). Among patients with Ct ≤ 30, 38 (27.5%) patients died, compared to the group of patients with Ct > 30 (14/100, 14.0%, *P* ═ 0.017). Other clinical and epidemiological parameters did not statistically differ between patients with lower and higher viral load (Table 3).

**Table 3:** Demographics, chronic diseases, clinical characteristics, and outcomes of COVID-19 patients regarding the viral load in nasopharyngeal swabs

**Characteristics of patients, *n* ═ 238**		**Ct ≤ 30, *n* (%)**	**Ct > 30, *n* (%)**	***P* value**
Nasopharingeal swab		138 (58.0)	100 (42.0)	**0.014**
Age (years)	18–44	7 (5.1)	5 (5.0)	0.168
	45–64	28 (20.3)	30 (30.0)	
	65–74	33 (23.9)	28 (28.0)	
	>75	70 (50.7)	37 (37.0)	
Gender	Male	85 (61.6)	63 (63.0)	0.893
Comorbidities	Yes	108 (78.3)	77 (77.0)	0.817
Immunocompromised	Yes	42 (30.4)	18 (18.0)	0.034*
Vaccination	Yes	32 (25.6)	33 (34.0)	0.171
Feces, *n* ═ 75	Positive	31 (63.3)	16 (64.0)	1.000*
Antibodies	Positive	33 (23.9)	51 (51.0)	**<0.001**
Fever, *n* ═ 233	>38 ^∘^C	54 (39.0)	36 (37.5)	0.201
Pneumonia	Yes	123 (89.1)	90 (90.0)	1.000*
ARI	Yes	102 (73.9)	67 (67.0)	0.251*
Oxygen support, *n* ═ 216	≥7L/min	50 (38.5)	25 (29.1)	0.156
Corticosteroids	Yes	107 (77.5)	73 (73.0)	0.421
ICU	Yes	23 (16.7)	10 (10.0)	0.184*
Disease severity	Moderate	37 (28.6)	34 (34.0)	0.253
	Severe/Critical	101 (73.2)	66 (66.0)	
Death outcome	Yes	38 (27.5)	14 (14.0)	0.017*

*Fisher exact test; Bold values represent statistical significance. COVID-19: Coronavirus disease 2019; ARI: Acute respiratory insufficiency; ICU: Intensive care unit; Ct: Cycle threshold.

**Table 4:** Demographics, chronic diseases, clinical characteristics, and outcomes of unvaccinated COVID-19 patients regarding the antibody status

**Characteristics of patients, *n* ═ 168**		**Antibody titer < 1, *n* (%)**	**Antibody titer ≥ 1, *n* (%)**	***P* value**
Antibodies		123 (73.2)	45 (26.8)	**0.005**
Age (years)	18–44	10 (8.1)	1 (2.2)	0.499
	45–64	31 (25.2)	13 (28.9)	
	65–74	29 (23.6)	13 (28.9)	
	>75	53 (43.1)	18 (40.0)	
Gender	Male	73 (59.3)	27 (60.0)	0.939
Comorbidities	Yes	88 (71.5)	36 (80.0)	0.325*
Immunocompromised	Yes	27 (22.0)	11 (24.4)	0.835*
Nasopharingeal swab, *n* ═ 161	≤30 Ct	69 (59.0)	28 (60.2)	0.718
	>30 Ct	48 (41.0)	16 (39.8)	
Feces, *n* ═ 47	Positive	20 (55.6)	9 (64.3)	0.752*
Fever, *n* ═ 165	>38 ^∘^C	48 (39.0)	16 (38.1)	0.915
Pneumonia	Yes	114 (92.7)	42 (93.3)	1.000*
ARI	Yes	96 (78.0)	27 (60.0)	0.029*
Oxygen support, *n* ═ 151	≥7L/min	48 (41.4)	10 (25.6)	0.234*
Corticosteroids	Yes	100 (81.3)	30 (66.7)	0.060*
ICU	Yes	22 (17.9)	2 (4.4)	0.026*
Disease severity	Moderate	29 (23.6)	14 (31.1)	0.325*
	Severe/Critical	94 (76.4)	31 (68.9)	
Death outcome	Yes	25 (20.3)	8 (17.8)	0.828*

*Fisher exact test; Bold values represent statistical significance. COVID-19: Coronavirus disease 2019; ARI: Acute respiratory insufficiency; ICU: Intensive care unit; Ct: Cycle threshold.

### The influence of previous infection on the clinical characteristics and outcomes of COVID-19

To examine the influence of previous infection, we stratified unvaccinated patients according to the SARS-CoV-2 seropositivity. Unvaccinated seronegative patients had a higher incidence of ARI (96/123, 78%; *P* ═ 0.029) and ICU admission (22/123, 17.9%, *P* ═ 0.026). No statistically significant difference was observed when other clinical and epidemiological parameters were compared between seropositive and seronegative patients (Table 4).

### The role of vaccination in acquiring antiviral protection

Our next aim was to determine the impact of positive antibody status on the clinical characteristics and outcomes of COVID-19 in vaccinated, as well as unvaccinated patients. Among the patients with positive IgGs, 41 (47.7%) were vaccinated and 45 (52.3%) were unvaccinated (*P* ═ 0.666), with no statistical difference regarding clinical manifestations or mortality. An observed trend indicated that unvaccinated patients exhibited an increased propensity for developing pneumonia and more severe disease manifestations, although this difference did not reach statistical significance (*P* ═ 0.107) (Table 5).

**Table 5:** Demographics, chronic diseases, clinical characteristics, and outcomes of COVID-19 patients with positive anti SARS CoV-2 IgGs regarding the vaccination status

**Characteristics of patients, *n* ═ 86**		**Vaccinated, *n* (%)**	**Unvaccinated, *n* (%)**	***P* value**
Vaccination		41 (47.7)	45 (52.3)	0.666
Age (years)	18–44	3 (7.3)	1 (2.2)	0.301
	45–64	7 (17.1)	13 (28.9)	
	65–74	17 (41.5)	13 (28.9)	
	>75	14 (34.1)	18 (40.0)	
Gender	Male	30 (73.2)	27 (60.0)	0.197
Comorbidities	Yes	36 (87.8)	36 (80.0)	0.390*
Immunocompromised	Yes	7 (17.1)	11 (24.4)	0.438*
Nasopharingeal swab, *n=*82	≤30 Ct	12 (30.0)	19 (45.2)	0.236
	>30 Ct	28 (70.0)	23 (54.8)	
Feces, *n* ═ 27	Positive	8 (61.5)	10 (71.4)	0.695
Fever, *n* ═ 81	>38 ^∘^C	14 (35.9)	16 (38.1)	0.920
Pneumonia	Yes	33 (80.5)	42 (93.3)	0.107*
ARI	Yes	26 (63.4)	27 (60.0)	0.826*
Oxygen support, *n* ═ 70	≥7L/min	10 (25.6)	10 (25.6)	0.556
Corticosteroids	Yes	27 (65.9)	30 (66.7)	0.091
ICU	Yes	7 (17.1)	2 (4.4)	0.080*
Disease severity	Moderate	17 (41.5)	14 (31.1)	0.220*
	Severe/Critical	24 (58.8)	31 (68.9)	
Death outcome	Yes	8 (19.5)	8 (17.8)	0.836

*Fisher exact test. COVID-19: Coronavirus disease 2019; SARS-CoV-2: Severe acute respiratory syndrome coronavirus 2; ARI: Acute respiratory insufficiency; ICU: Intensive care unit; Ct: Cycle threshold.

Seropositive patients were stratified according to the antibody titers into high/low groups. A moderate positive correlation between a high antibody titer and higher Ct values was observed (*P* ═ 0.008), while other variables did not show a correlation with the antibody titer (Table S3).

### The influence of SARS-CoV-2 RNA feces excretion on the clinical characteristics and outcomes of COVID-19

Forty-seven patients (62.7%) were positive for SARS-CoV-2 RNA (*P* ═ 0.028) out of 75 patients who provided a feces sample. There were no statistical differences in vaccination status (15/47, 31.9%, *P* ═ 0.493), antibody status (18/47, 38.3%, *P* ═ 0.628), or death outcome (5/47, 10.6%, *P* ═ 0.706) between fecal SARS-CoV-2 RNA positive and negative groups of patients regarding the clinical outcome of COVID-19. Furthermore, there were no differences in age (*P* ═ 0.684), gender (male 26/47, 55.3%, *P* ═ 0.138), or presence of comorbidities (34/47, 72.3%, *P* ═ 1.000) between fecal SARS-CoV-2 RNA positive and negative groups of patients. Similarly, there were no differences regarding the occurrence of pneumonia (41/47, 87.2%, *P* ═ 1.000), ARI (33/47, 70.2%, *P* ═ 0.617), nor ICU admission (9/47, 19.1%, *P* ═ 0.080) between fecal SARS-CoV-2 RNA positive and negative group of patients (Table 6).

**Table 6:** Demographic characteristics, chronic disease presence, clinical characteristics, and outcomes of COVID-19 patients with SARS-CoV-2 positive feces samples

**Characteristics of patients, *n* ═ 75**		**Positive feces samples, *n* (%)**	**Negative feces samples, *n* (%)**	***P* value**
Feces		47 (62.7)	28 (37.3)	0.028
Age (years)	18–44	4 (8.5)	2 (7.1)	0.684
	45–64	13 (27.7)	11 (39.3)	
	65–74	11 (23.4)	7 (25.0)	
	>75	19 (40.4)	8 (28.6)	
Gender	Male	26 (55.3)	21 (75.0)	0.138
Comorbidities	Yes	34 (72.3)	21 (75.0)	1.000
Immunocompromised	Yes	8 (17.0)	7 (25.0)	0.552*
Vaccination, *n* ═ 66	Vaccinated	15 (31.9)	6 (21.4)	0.493
Nasopharingeal swab, *n* ═ 71	≤30 Ct	31 (66.0)	19 (67.9)	1.000
	>30 Ct	16 (34.0)	9 (32.1)	
Antibodies	Positive	18 (38.3)	9 (32.1)	0.628
Fever	> 38 ^∘^C	24 (51.1)	13 (46.4)	0.812
Pneumonia	Yes	41 (87.2)	24 (85.7)	1.000*
ARI	Yes	33 (70.2)	18 (64.3)	0.617*
Oxygen support, *n* ═ 63	≥7L/min	13 (33.3)	8 (33.3)	0.100
Corticosteroids	Yes	37 (78.7)	18 (64.3)	0.188
ICU	Yes	9 (19.1)	1 (3.6)	0.080*
Disease severity	Moderate	14 (29.8)	10 (35.7)	0.614
	Severe/Critical	33 (70.2)	18 (64.3)	
Death outcome	Yes	5 (10.6)	2 (7.14)	0.706

*Fisher exact test. COVID-19: Coronavirus disease 2019; SARS-CoV-2: Severe acute respiratory syndrome coronavirus 2; ARI: Acute respiratory insufficiency; ICU: Intensive care unit; Ct: Cycle threshold.

## Discussion

We included 251 patients with manifested COVID-19 requiring hospital treatment in this study. The results indicated that SARS-CoV-2 IgGs were more frequently present in patients with lower viral load and in immunocompetent patients. Unvaccinated patients without previous SARS-CoV-2 infection had a higher incidence of ARI as well as ICU admission, while other clinical and epidemiological parameters, as well as mortality rate, did not show a statistically significant difference between patients with positive or negative antibody titers in this group. Patients with positive antibody titers were similarly distributed between those who had been vaccinated and those who had resolved COVID-19, with no statistical difference regarding clinical manifestations or death outcomes. Although the difference was not statistically significant, unvaccinated patients with positive antibody titers were more likely to develop pneumonia and more severe disease compared to unvaccinated patients with negative antibody titers. Finally, there were no differences in age, gender, vaccination status, comorbidities, and death outcome depending on SARS-CoV-2 RNA presence in feces samples.

In line with previous findings, our study further confirms that older age is the primary risk factor for mortality, followed by ICU admission, immunocompromised status, and disease severity [19].

We used Ct values as an indicator of a viral load in each tested sample in this study. The Ct value could indicate potential infectivity [20]. We found higher levels of SARS-CoV-2-specific antibodies, indicating the presence of previously acquired neutralizing anti-spike SARS-CoV-2 antibodies as a result of vaccination or resolved infections in patients with lower viral loads [10]. Furthermore, immunocompromised patients exhibited a higher viral load compared to their immunocompetent counterparts, likely due to a diminished immune response to the infection [21].

Taking into account the time of our research conduction, we assume that the dominant variant in the study was the Omicron variant of SARS-CoV-2 [22, 23]. It is well established that the Omicron variant has developed several defense mechanisms against the immunity acquired by a previous infection [24, 25] or vaccination, compared to the previous SARS-CoV-2 variants [26].

Unvaccinated patients with a negative antibody status upon hospital admission demonstrated an increased likelihood of developing ARI and requiring ICU admission. These findings underscore the critical role of vaccination, particularly for individuals with no prior exposure to SARS-CoV-2. Additionally, the results revealed no significant difference in patients with a positive titer of specific IgGs, regardless of vaccination status.

Within the initial weeks following vaccination, patients who receive the vaccine exhibit milder clinical manifestations compared to those unvaccinated when contracting COVID-19 [27, 28]. Similarly, prior vaccination provides benefits for hospitalized patients [28–30]. Notably, the patients included in this study were vaccinated with only two doses of the COVID vaccine, although three doses of the vaccine were recommended by the WHO during the study period [31]. Furthermore, most of our patients received a second dose of the vaccine five or more months before infection occurred. In addition, the lack of the third dose administration among patients in this study could also affect the effectiveness of vaccination against the dominant variants of SARS-CoV-2 during the study period. Also, a possible explanation for the lack of greater advantage of the COVID-19 vaccine observed in this study could be that mostly the elderly with comorbidities and immunocompromised people were enrolled, rather than immunocompetent younger people [17, 32, 33].

Our recent study demonstrated that previous contact with SARS-CoV-2, irrespective of vaccination, has a protective effect during subsequent infection, indicating that immunity acquired by previous infection might play an essential role in the prevention of symptomatic disease, especially the appearance of high fever, and loss of taste and smell [17]. This could be explained by the stronger mucosal immunity that arises as a result of a natural infection. Available COVID-19 vaccines are most often administered intramuscularly and primarily elicit IgG antibody response, with a weak response of mucosal IgA antibodies, which have a superior antiviral effect at the entry points of infection [34–36]. Therefore, hybrid immunity gained by vaccination and infection creates a better immune defense, with circulating IgG antibodies and mucosal IgA antibodies [37].

Previous research has shown that 40%–60% of COVID-19 patients have a positive feces sample for SARS-CoV-2. Furthermore, SARS-CoV-2 RNA could be detected in feces even after the negativization of NP swabs [16, 38]. Also, the presence of viral particles in the tissue of the gastrointestinal tract suggests acute viral replication [39] and potential for fecal–oral transmission of the virus [40].

One of the questions of this research was the correlation between SARS-CoV-2 RNA-positive feces samples and clinical manifestations of COVID-19. There is conflicting evidence on the association between a positive feces PCR test for SARS-CoV-2 RNA and the severity of the patient’s clinical manifestation [41, 42], but several previous studies have shown that there is no significant correlation between the detection of the virus in the feces and the severity of clinical manifestations [38, 43]. However, the presence of viral particles in the feces does not necessarily mean that the virus can be transmitted via the feces. Our results are in line with previous research, showing no difference in the clinical manifestations, course of the disease, or outcome in patients who tested positive for SARS-CoV-2 RNA in feces, compared to patients with negative SARS-CoV-2 RNA results in feces samples.

This study has several limitations. The study only included hospitalized patients, and as such, the results cannot be extrapolated to the overall population infected by SARS-CoV-2. All vaccinated patients received only two doses of vaccine in the study period which might affect the vaccine effectiveness against the Omicron variant. Furthermore, the Omicron variant was presumed rather than identified in each study participant. However, the study period overlaps with the peak of the COVID-19 prevalence in Bosnia and Herzegovina, as well as the Omicron predominance during the pandemic. Some of the observed trends in our results were not statistically significant due to the small number of participants. We were not able to detect previously recovered asymptomatic COVID-19 among investigated patients based on the anamnestic records and SARS-CoV-2 IgG detection method used in our study. Since the detection of SARS-CoV-2 IgGs in our study did not present neutralizing capacity, further study to clarify this issue should be conducted.

## Conclusion

In this study, we showed that hospitalized unvaccinated patients without previous infection had a higher incidence of ARI and ICU admission. Despite the significant presence of comorbidities among vaccinated patients, ARI and pneumonia occurred to a lower extent compared to unvaccinated patients. Given that these were hospitalized patients, the impact of vaccination and the presence of antibodies did not affect the clinical outcome of COVID-19. Fecal excretion of SARS-CoV-2 RNA had no impact on the clinical outcome of COVID-19. Moreover, in one-third of patients with SARS-CoV-2-positive NP swabs, SARS-CoV-2 RNA was not detected in the feces. Thus, caution should be taken when using feces as a diagnostic sample for detecting SARS-CoV-2 infection.

## Supplemental data

**Table S1:** Demographics, chronic diseases, clinical characteristic, and outcomes of COVID-19 patients regarding the death outcome

**Characteristics of patients, *n* ═ 251**		**Yes, *n* (%)**	**No, *n* (%)**	***P* value**
Death		53 (21.1)	198 (78.9)	0.005
Age (years)	18-44	0 (0)	14 (7.1)	**0.001**
	45–64	6 (11.3)	54 (27.3)	
	65–74	11 (20.8)	55 (27.8)	
	>75	36 (67.9)	78 (37.9)	
Gender	Male	36 (67.9)	118 (59.6)	0.341*
Comorbidities	Yes	46 (86.8)	151 (76.3)	0.131*
Immunocompromised	Yes	19 (35.8)	44 (22.2)	0.050*
Vaccination, *n* ═ 234	Vaccinated	10 (19.2)	56 (30.8)	0.118*
Nasopharingeal swab, *n* ═ 238	≤ 30 Ct	35 (67.3)	103 (55.4)	0.153
	> 30 Ct	17 (32.7)	83 (44.6)	
Feces, *n* ═ 75	Positive	5 (71.4)	42 (61.8)	0.706*
Antibodies	Positive	16 (30.2)	72 (36.4)	0.403
Fever, *n* ═ 246	>38 ^∘^C	15 (71.2)	77 (39.7)	0.196*
Pneumonia	Yes	51 (96.2)	175 (88.4)	0.121*
ARI	Yes	46 (86.8)	132 (66.7)	0.004*
Oxygen support, *n* ═ 226	≥7L/min	20 (41.7)	62 (34.8)	0.382
Corticosteroids	Yes	43 (81.1)	146 (73.7)	0.370*
ICU	Yes	8 (15.1)	27 (13.6)	0.824*
Disease severity	Moderate	7 (13.2)	68 (34.3)	0.002*
	Severe/Critical	46 (86.8)	130 (65.7)	

*Fisher exact test; Bold values represent statistical significance. COVID-19: Coronavirus disease 2019; ARI: Acute respiratory insufficiency; ICU: Intensive care unit; Ct: Cycle threshold.

**Table S2:** Demographics, chronic diseases, clinical characteristics, and outcomes of COVID-19 patients regarding the vaccination type

**Characteristics of patients, *n* ═ 66**		**Unknown, *n* (%)**	**mRNA vaccine, *n* (%)**	**Adenoviral vaccine, *n* (%)**	**Whole inactivated vaccine, *n* (%)**	***P* value**
Vaccination	Yes	14 (21.2)	23 (34.8)	15 (22.7)	14 (21.2)	0.327
Age (years)	18–44	0 (0.0)	2 (8.7)	0 (0.0)	1 (7.7)	**0.045**
	45–64	1 (7.1)	7 (30.4)	1 (6.7)	2 (14.3)	
	65–74	1 (7.1)	8 (34.8)	7 (46.7)	5 (35.7)	
	>75	12 (85.7)	6 (26.1)	7 (46.7)	6 (42.9)	
Gender	Male	5 (35.7)	21 (91.3)	12 (80.0)	9 (64.3)	**0.003**
Comorbidities	Yes	14 (100.0)	20 (87.0)	15 (100.0)	12 (85.7)	0.235
Immunocompromised	Yes	5 (35.7)	6 (26.1)	5 (33.3)	3 (21.4)	0.818
Ct value	≤30	8 (57.1)	11 (47.8)	7 (46.7)	6 (42.9)	0.891
	>30	6 (42.9)	12 (52.2)	8 (53.3)	8 (57.1)	
Feces, *n* ═ 21	Yes	5 (100)	1 (25.0)	4 (66.7)	4 (66.7)	0.131
Antibodies	Yes	6 (42.9)	15 (65.2)	11 (73.3)	9 (64.3)	0.372
Symptoms	Yes	12 (85.7)	22 (95.7)	15 (100.0)	13 (92.9)	0.427
Fever	>38 ^∘^C	2 (14.3)	9 (39.1)	7 (46.7)	6 (42.9)	0.508
Pneumonia	Yes	11 (78.6)	16 (69.6)	14 (93.3)	13 (92.9)	0.179
ARI	Yes	8 (57.1)	12 (52.2)	13 (86.7)	9 (64.3)	0.172
Oxygen support	≥7L/min	1 (7.1)	4 (17.4)	8 (53.3)	3 (21.4)	0.069
Corticosteroids	Yes	9 (64.3)	13 (56.5)	13 (86.7)	9 (64.3)	0.280
ICU	Yes	0 (0.0)	2 (8.7)	4 (26.7)	2 (14.3)	0.156
Disease severity	Moderate	6 (42.9)	14 (60.9)	2 (13.3)	6 (42.9)	0.058
	Severe/Critical	8 (57.1)	9 (39.1)	13 (86.7)	8 (57.1)	
Death outcome	Yes	5 (35.7)	5 (21.7)	3 (20.0)	2 (14.3)	0.575*

*Fisher exact test; Bold values represent statistical significance. COVID-19: Coronavirus disease 2019; Ct: Cycle threshold; ARI: Acute respiratory insufficiency; ICU: Intensive care unit.

**Table S3:** Correlation matrix between high and low antibody titer and clinical and epidemiological data

		**1**	**2**	**3**	**4**	**5**	**6**	**7**	**8**	**9**	**10**	**11**	**12**	**13**	**14**	**15**	**16**
1	Antibody titer																
2	Age	−0.055															
3	Gender	−0.099	0.276**														
4	Comorbidites	−0.026	0.239*	0.256*													
5	Immunocompromised	−0.094	−0.034	0.076	0.228*												
6	Nasopharyngeal swab	0.281**	−0.107	−0.099	−0.09	−0.264*											
7	Feces	0.183	0.158	0.047	−0.085	−0.035	0.077										
8	Vaccination	−0.219*	0.053	0.209	−0.069	0.124	−0.219*	0.039									
9	Fever	0.071	−0.327**	−0.215*	0.027	0.007	0.033	−0.369**	0.041								
10	Pneumonia	0.053	0.219*	0.063	0.023	0.198	0.053	0.128	0.199	0.064							
11	ARI	−0.15	0.157	0.048	−0.026	0.076	−0.054	−0.028	−0.046	−0.006	0.335**						
12	Oxygen support	−0.017	0.096	0.059	0.031	0.179	−0.2	0.03	−0.073	−0.055	0.296**	0.605**					
13	Corticosteroids	−0.06	0.264*	0.112	0.026	0.015	−0.06	−0.017	0.051	0.021	0.247*	0.784**	0.467**				
14	ICU	−0.117	−0.149	0.144	0.147	0.005	−0.117	0.015	−0.209	0.113	0.014	0.191	0.329**	0.077			
15	Disease severity	−0.115	0.156	0.013	−0.006	0.052	−0.018	−0.041	0.079	0.193	0.286**	0.807**	0.424**	0.626**	0.177		
16	Death outcome	0.072	0.17	−0.101	0.205	0.182	−0.11	0.145	−0.05	−0.169	0.089	0.132	0.105	0.08	−0.062	0.111	

**Correlation is significant at the 0.01 level (2-tailed). *Correlation is significant at the 0.05 level (2-tailed); ARI: Acute respiratory insufficiency; ICU: Intensive care unit.

## References

[1] CDC. https://www.cdc.gov/coronavirus/2019-ncov/variants/variant-classifications.html.

[2] Bermejo-Martin JF, González-Rivera M, Almansa R, Micheloud D, Tedim AP, Domínguez-Gil M (2020). Viral RNA load in plasma is associated with critical illness and a dysregulated host response in COVID-19. Crit Care.

[3] Gandhi RT, Lynch JB, Del Rio C (2020). Mild or moderate Covid-19. N Engl J Med.

[4] Dadras O, Afsahi AM, Pashaei Z, Mojdeganlou H, Karimi A, Habibi P (2022). The relationship between COVID-19 viral load and disease severity: a systematic review. Immun Inflamm Dis.

[5] Walsh KA, Jordan K, Clyne B, Rohde D, Drummond L, Byrne P (2020). SARS-CoV-2 detection, viral load and infectivity over the course of an infection. J Infect.

[6] Wang X, Zheng J, Guo L, Yao H, Wang L, Xia X (2020). Fecal viral shedding in COVID-19 patients: Clinical significance, viral load dynamics and survival analysis. Virus Res.

[7] Miller TE, Garcia Beltran WF, Bard AZ, Gogakos T, Anahtar MN, Astudillo MG (2020). Clinical sensitivity and interpretation of PCR and serological COVID-19 diagnostics for patients presenting to the hospital. FASEB J.

[8] Scourfield DO, Reed SG, Quastel M, Alderson J, Bart VMT, Teijeira Crespo A (2021). The role and uses of antibodies in COVID-19 infections: a living review. Oxf Open Immunol.

[9] Long Q-X, Liu B-Z, Deng H-J, Wu G-C, Deng K, Chen Y-K (2020). Antibody responses to SARS-CoV-2 in patients with COVID-19. Nat Med.

[10] Galipeau Y, Greig M, Liu G, Driedger M, Langlois M-A (2020). Humoral responses and serological assays in SARS-CoV-2 infections. Front Immunol.

[11] Castro Dopico X, Ols S, Loré K, Karlsson Hedestam GB (2022). Immunity to SARS-CoV-2 induced by infection or vaccination. J Intern Med.

[12] Anjos D, Fiaccadori FS, Servian C do P, da Fonseca SG, Guilarde AO, Borges MASB (2022). SARS-CoV-2 loads in urine, sera and stool specimens in association with clinical features of COVID-19 patients. J Clin Virol Plus.

[13] Cuicchi D, Lazzarotto T, Poggioli G (2021). Fecal-oral transmission of SARS-CoV-2: review of laboratory-confirmed virus in gastrointestinal system. Int J Colorectal Dis.

[14] Wong MC, Huang J, Lai C, Ng R, Chan FKL, Chan PKS (2020). Detection of SARS-CoV-2 RNA in fecal specimens of patients with confirmed COVID-19: a meta-analysis. J Infect.

[15] Cerrada-Romero C, Berastegui-Cabrera J, Camacho-Martínez P, Goikoetxea-Aguirre J, Pérez-Palacios P, Santibáñez S (2022). Excretion and viability of SARS-CoV-2 in feces and its association with the clinical outcome of COVID-19. Sci Rep.

[16] Daou M, Kannout H, Khalili M, Almarei M, Alhashami M, Alhalwachi Z (2022). Analysis of SARS-CoV-2 viral loads in stool samples and nasopharyngeal swabs from COVID-19 patients in the United Arab Emirates. PLoS One.

[17] Laura L, Dalmatin-Dragišić M, Martinović K, Tutiš B, Herceg I, Arapović M (2023). Does pre-existing immunity determine the course of SARS-CoV-2 infection in health-care workers? Single-center experience. Infection.

[18] Šušak B, Mikulić V, Lazarević A, Mikulić I, Arapovic J (2022). Sustained seroprevalence of SARS-CoV-2 antibodies one year after infection: one of the first COVID-19 cluster cases in Bosnia and Herzegovina. Bosn J Basic Med Sci.

[19] Tabatabai M, Juarez PD, Matthews-Juarez P, Wilus DM, Ramesh A, Alcendor DJ (2023). An analysis of COVID-19 mortality during the dominancy of alpha, delta, and Omicron in the USA. J Prim Care Community Health.

[20] Platten M, Hoffmann D, Grosser R, Wisplinghoff F, Wisplinghoff H, Wiesmüller G, et al.

[21] Agrati C, Bartolini B, Bordoni V, Locatelli F, Capobianchi MR, Di Caro A (2023). Emerging viral infections in immunocompromised patients: a great challenge to better define the role of immune response. Front Immunol.

[22] Mohapatra RK, Sarangi AK, Kandi V, Azam M, Tiwari R, Dhama K (2022). Omicron (B.1.1.529 variant of SARS-CoV-2); an emerging threat: current global scenario. J Med Virol.

[23] Araf Y, Akter F, Tang Y-D, Fatemi R, Parvez MSA, Zheng C (2022). Omicron variant of SARS-CoV-2: genomics, transmissibility, and responses to current COVID-19 vaccines. J Med Virol.

[24] Schubert M, Bertoglio F, Steinke S, Heine PA, Ynga-Durand MA, Maass H (2022). Human serum from SARS-CoV-2-vaccinated and COVID-19 patients shows reduced binding to the RBD of SARS-CoV-2 Omicron variant. BMC Med.

[25] Danza P, Koo TH, Haddix M, Fisher R, Traub E, OYong K (2022). SARS-CoV-2 infection and hospitalization among adults aged ≥18 years, by vaccination status, before and during SARS-CoV-2 B.1.1.529 (Omicron) variant predominance - Los Angeles County, California, November 7, 2021–January 8, 2022. MMWR Morb Mortal Wkly Rep.

[26] Callaway E (2021). Omicron likely to weaken COVID vaccine protection. Nature.

[27] Antonelli M, Penfold RS, Merino J, Sudre CH, Molteni E, Berry S (2022). Risk factors and disease profile of post-vaccination SARS-CoV-2 infection in U.K. users of the COVID symptom study app: a prospective, community-based, nested, case-control study. Lancet Infect Dis.

[28] Basnet A, Tamang B, Pokhrel N, Khadka S, Shrestha MR, Ghimire S (2022). First-generation SARS-CoV-2 vaccines: a comparative analysis between vaccinated and unvaccinated hospitalized patients infected with SARS–CoV–2. Kathmandu Univ Med J (KUMJ).

[29] Rearte A, Castelli JM, Rearte R, Fuentes N, Pennini V, Pesce M (2022). Effectiveness of rAd26-rAd5, ChAdOx1 nCoV-19, and BBIBP-CorV vaccines for risk of infection with SARS-CoV-2 and death due to COVID-19 in people older than 60 years in Argentina: a test-negative, case-control, and retrospective longitudinal study. Lancet.

[30] Desai A, Desai P, Mehta J, Sachora W, Bharti N, Patel T (2021). Measuring the impact of a single dose of ChAdOx1 nCoV-19 (recombinant) coronavirus vaccine on hospital stay, ICU requirement, and mortality outcome in a tertiary care centre. Int J Infect Dis.

[31] Interim statement on booster doses for COVID-19 vaccination [Internet]. https://www.who.int/news/item/22-12-2021-interim-statement-on-booster-doses-for-covid-19-vaccination/-/-update-22-december-2021.

[32] Lin W, Xie Z, Li Y, Li L, Wen C, Cao Y (2021). Association between detectable SARS-COV-2 RNA in anal swabs and disease severity in patients with coronavirus disease 2019. J Med Virol.

[33] Çavuş SA, Çelik M, Irmak Ç, Helvacı G, Ömeroğlu Şimşek G, Coşkun F (2023). Vaccination status and outcome of hospitalized patients with coronavirus disease 2019 before and after the spread of omicron variant: an observational study from İzmir, Turkey. Thorac Res Pract.

[34] Ewer KJ, Barrett JR, Belij-Rammerstorfer S, Sharpe H, Makinson R, Morter R (2021). T cell and antibody responses induced by a single dose of ChAdOx1 nCoV-19 (AZD1222) vaccine in a phase 1/2 clinical trial. Nat Med.

[35] Focosi D, Maggi F, Casadevall A (2022). Mucosal vaccines, sterilizing immunity, and the future of SARS-CoV-2 virulence. Viruses.

[36] Wang Z, Lorenzi JCC, Muecksch F, Finkin S, Viant C, Gaebler C (2021). Enhanced SARS-CoV-2 neutralization by dimeric IgA. Sci Transl Med.

[37] Vicenti I, Gatti F, Scaggiante R, Boccuto A, Zago D, Basso M (2022). The second dose of the BNT162b2 mRNA vaccine does not boost SARS-CoV-2 neutralizing antibody response in previously infected subjects. Infection.

[38] Chen Y, Chen L, Deng Q, Zhang G, Wu K, Ni L (2020). The presence of SARS-CoV-2 RNA in the feces of COVID-19 patients. J Med Virol.

[39] Qian Q, Fan L, Liu W, Li J, Yue J, Wang M (2021). Direct evidence of active SARS-CoV-2 replication in the intestine. Clin Infect Dis.

[40] Xiao F, Sun J, Xu Y, Li F, Huang X, Li H (2020). Infectious SARS-CoV-2 in feces of patient with severe COVID-19. Emerg Infect Dis.

[41] Hossain B, Malik F, Khan A, Abidi M, Marhaba A, Oranu A (2023). Prevalence and impact of gastrointestinal manifestations in COVID-19 patients: a systematic review. J Commun Hosp Intern Med Perspect.

[42] Natarajan A, Zlitni S, Brooks EF, Vance SE, Dahlen A, Hedlin H (2022). Gastrointestinal symptoms and fecal shedding of SARS-CoV-2 RNA suggest prolonged gastrointestinal infection. Med.

[43] Wu W, Shi D, Zhu X, Xie J, Xu X, Chen Y (2022). Characteristics of COVID-19 patients with SARS-CoV-2 positivity in feces. Front Cell Infect Microbiol.

